# The Cross Talk between cGMP Signal Pathway and PKC in Pulmonary Endothelial Cell Angiogenesis

**DOI:** 10.3390/ijms150610185

**Published:** 2014-06-06

**Authors:** Zhen Zeng, Ying-Chuan Li, Zhi-Hua Jiao, Jun Yao, Ying Xue

**Affiliations:** Department of Anesthesiology, Shanghai Jiao Tong University Affiliated Sixth People’s Hospital, Shanghai 200233, China; E-Mails: daisycui0128@126.com (Y.-C.L.); zz73cxf@sohu.com (Z.-H.J.); 18930173652@189.cn (J.Y.); zlxsh4808@163.com (Y.X.)

**Keywords:** cellular proliferation, pulmonary vasular endothelial cell, angiogenesis, cGMP (cyclic guanosine monophosphate), PKC (protein kinase C)

## Abstract

Angiogenic proliferation of vascular endothelial cells is believed to play an important role in pulmonary vascular remodeling in pulmonary arterial hypertension. In the present study, we found that c-GMP (cyclic guanosine monophosphate) inhibited the proliferation and tube formation of pulmonary vascular endothelial cells induced by TGF-β1, and that this process was reversed by PKG (protein kinase G) inhibitor and PKC (protein kinase C) inhibitor. In addition, small interfering RNA (siRNA) targeting ERK also reduced cellular proliferation. Furthermore, western blotting showed that cGMP down-regulated the phosphorylation level of ERK1/2, which was reversed not only by PKG inhibitor but also by PKC inhibitor. Silencing different PKC isoforms showed that PKCΔ, PKCγ and PKCα were involved in ERK phosphorylation, suggesting that PKC kinases have a permissive action. Three subtypes, PKCΔ, PKCγ and PKCα are likely to be involved the phosphorylation suppression of ERK included cGMP. Taken together, these data suggest that ERK phosphorylation mediates the proliferation of pulmonary vascular endothelial cells, and PKC kinases have a permissive action in this process.

## 1. Introduction

Severe pulmonary arterial hypertension (PAH), whether idiopathic or associated with known causes (secondary forms), may have persistent structural alterations of microscopically small pulmonary arterioles, *i.e.*, pulmonary vascular remodeling, which is believed to be caused by angiogenic proliferation of the pulmonary vascular endothelial cell (PVEC) [1,2]. The past few years have seen a remarkable increase in our knowledge of the cellular and molecular mechanisms responsible for the pathobiology of PAH.

It is believed that circulating endothelial progenitors (EPCs) play two major roles in the cardiovascular system: endothelial healing and neo-angiogenesis, which might have important implications in PAH. [3] It has been proved that sildenafil, a phosphodiesterase type 5 inhibitor, not only reduces pulmonary artery pressure (PAP) in clinical treatment [4,5], but also attenuates angiogenic proliferation of pulmonary vascular endothelial cell in the process of pulmonary vascular remodeling [6,7]. This anti-proliferative effect is related to the cGMP and ERK pathways [6,8]. Studies have also revealed that protein kinase C (PKC) expression is up-regulated during the development of pulmonary vascular remodeling [9]. Furthermore, it has also been shown that PKC plays an essential role in sildenafil-induced cardio-protection in rabbits, which demonstrates the relationship between PKC and the cGMP pathway [10]. Since tube formation assay can mimic the endothelial repair process after pulmonary vascular injuries *in vitro*, we wanted to know what functions cGMP and PKC perform in angiogenesis by endothelial cells derived from EPCs during the development of PAH.

We are interested in the possible relations of PKC expression and cGMP related anti-proliferative effect in pulmonary vascular remodeling. To elucidate the underlying mechanism, in the present study, we employed a PKC inhibitor and small interfering RNA (siRNA) targeting ERK and PKC to investigate whether there is some cross-talk between cGMP dependent angiogenesis and PKC in pulmonary vascular endothelial cell proliferation.

## 2. Results and Discussion

### 2.1. TGF-β1 Induces PEVC (Pulmonary Vascular Endothelial Cell) Proliferation

As an important member of the transforming growth factor beta superfamily of cytokines, TGF-β1 plays a crucial role in angiogenesis. However, studies show that TGF-β1 may induce pulmonary vascular remodeling in pulmonary hypertension animal models. We firstly examined its effect on PVEC cells *in vitro*. TGF-β1 increased PVEC cell proliferation in a concentration-dependent fashion. The optical density (OD) values in MTT (3-(4,5)-dimethylthiahiazo-(z-y1)-3,5-di-phenytetrazoliumromide) assay, which represent the number of viable cells or cell proliferation, were markedly increased after 24 h incubation with TGF-β1. And after 48 h incubation with TGF-β1 at 1, 10 and 100 ng/mL the OD values were increased from 0.73 in control cells to 1.11 ± 0.10, 1.37 ± 0.11 and 1.63 ± 0.19 (*p* < 0.05) respectively.

### 2.2. The Reversibility of Cell Proliferation by cGMP (Cyclic Guanosine Monophosphate)

**Figure 1:**
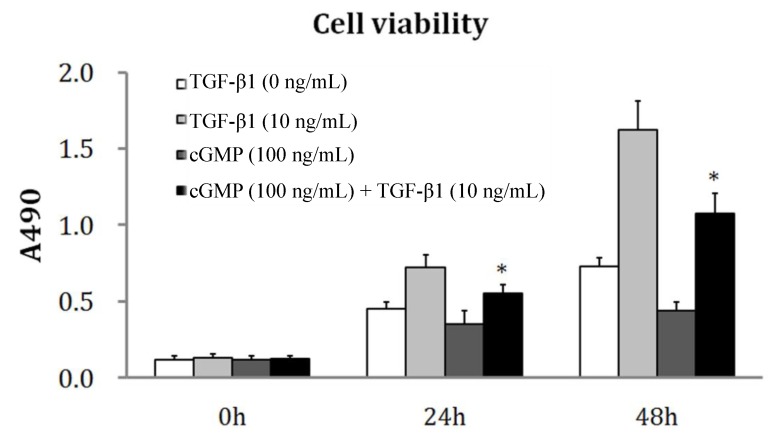
The effect of cGMP (cyclic guanosine monophosphate) on cellular proliferation. The OD values in MTT (3-(4,5)-dimethylthiahiazo-(z-y1)-3,5-di-phenytetrazoliumromide) assay were measured after PVEC (pulmonary vascular endothelial cell)cells were treated with fresh medium containing TGF-β1 (0, 10 ng/mL), cGMP (100 ng/mL) and TGF-β1 (100 ng/mL)+cGMP(10 ng/mL) for 24and 48 h. The OD values of cells were remarkably increased after 24 and 48 h treatments with TGF-β1. However, for the cells incubated with cGMP at 10 ng/mL and TGF-β1 at 100 ng/mL together after 24 and 48 h, the increased OD value was inhibited by 23% and 35%, compared with those incubated with TGF-β1 alone. * *p* < 0.05, *vs.* the group of 10 ng/mL TGF-β1.

The inhibitory effect of cGMP on cellular proliferation induced by TGF-β1 was examined in PVEC cells treated with TGF-β1 and cGMP together. There was no significant difference between the OD values of cells treated with cGMP and the control group. The proliferation of cells was remarkably increased after 24 and 48 h treatments with TGF-β1. But the proliferation was inhibited by incubation with cGMP at 10 ng/mL and TGF-β1 at 100 ng/mL together after 24 and 48 h (Figure 1).

### 2.3. The Effects of PKC (Protein Kinase C) Inhibitor on Cell Proliferation Regulated by the cGMP-PKG (Protein Kinase G) Pathway

**Figure 2:**
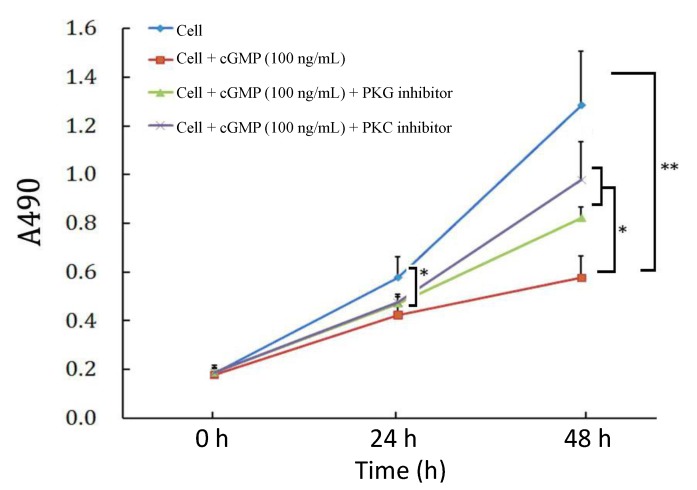
Effects of PKC and PKG inhibitors on cellular proliferation suppressed by cGMP. Cells were pretreated with the PKG inhibitor or PKC inhibitor for 2 h, and then were treated with cGMP for 24 or 48 h and subjected to MTT assay. The results showed that in comparison with the control group, the proliferative activity was reduced in the group treated with 100 ng/mL cGMP, with significant differences at 24 h (*p* < 0.05) and 48 h (*p* < 0.01); and the suppression was partially recovered by the PKG inhibitor and PKC inhibitor (*p* < 0.05, *vs.* the group treated with 100 ng/mL cGMP, 48 h), and the effect of PKG inhibitor was slightly stronger than that of PKC inhibitor, through there was no significant difference between these two groups. * *p* < 0.05 and ** *p* < 0.01.

As previously tested, cell proliferation was inhibited after 48 h incubated with cGMP. This effect can be partially reversed by the PKG inhibitor and PKC inhibitor. And the PKC inhibitor exhibited a slightly stronger effect than the PKG inhibitor (Figure 2).

### 2.4. The Reversibility of Cell Proliferation of ERK siRNA

**Figure 3:**
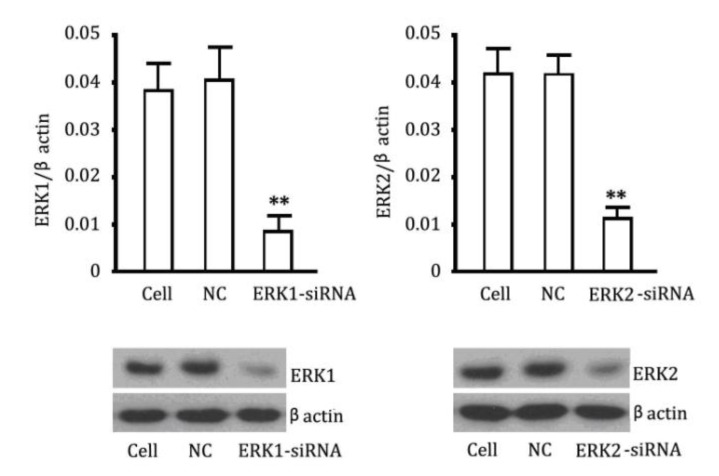
Knockdown of ERK1/2 by siRNAs. (**Upper**) Measurements of ERK1 and ERK2 expression levels in PEVCs (Pulmonary Vascular Endothelial Cells)by QRT-PCR; (**Lower**) Detection of ERK1 and ERK2 expression levels in PEVCs by western blotting. The negative control (NC) had no a interference. ** *p* < 0.01, *vs.* control.

**Figure 4:**
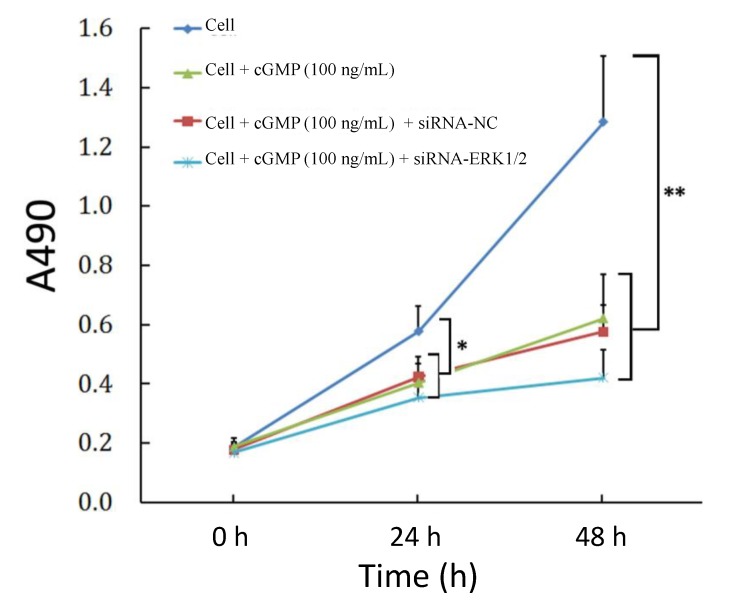
Effects of ERK block on cellular proliferation. Cells were incubated with 100 ng/mL cGMP or siRNA targeting ERK and MTT assay was performed 24 or 48 h later. The result showed that cGMP and ERK silencing both reduced the proliferative activity of microartery endothelial cells, with a significant difference at 24 and 48 h (*p* < 0.05 or 0.01, *vs.* control group), through there was no significant difference between these two groups. * *p* < 0.05 and ** *p* < 0.01, *vs.* control group.

Since a previous study suggests that ERK may be involved in the proliferation of pulmonary vascular endothelial cells, [6], we also investigated the proliferation in cells treated with small interfering RNA targeting ERK. We firstly verified ERK1/2 knockdown by chemically synthesized siRNAs. As shown in Figure 3, siRNAs targeting ERK1 and ERK2 respectively decreased significantly their mRNA levels (*p* < 0.01, *vs.* the control). Western blotting results showed similar changes in ERK1 and ERK2 protein levels in cells after treatment with siRNAs targeting ERK1 and ERK2. Then we used these siRNA to treat PVEC(pulmonary vascular endothelial cells) to evaluate the effects of ERK block on cellular proliferation. The OD values of PVEC cells were considerably more decreased when compared to the data of the group treated with cGMP, though there was no significant difference between these two groups (Figure 4).

### 2.5. Cross Talk Effects between PKC Inhibitor and cGMP Down-Regulated ERK Pathway

**Figure 5:**
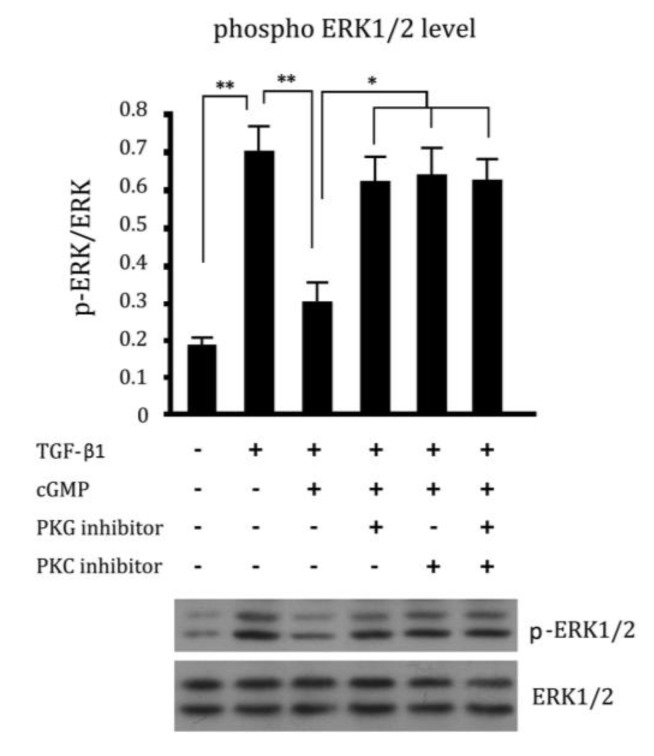
Cross talk effects between PKC inhibitor and cGMP pathway. The concentrations of ERK1/2 were expressed as gray scale. The phosphorylation levels of ERK1/2 were expressed as the ratio of gray scale of p-ERK/ERK. The phosphorylation level of ERK1/2 was remarkably increased in cells incubated with TGF-β1. The increased phosphorylation level of ERK1/2 was down-regulated by cGMP in 10 ng/mL. And the effect of decreased phosphorylation level of ERK1/2 was abrogated by PKG inhibitor and PKC inhibitor. ** *p* < 0.01 and * *p* < 0.05.

The phosphorylation level of ERK1/2, which was closely related to the proliferation of cells, was examined by western blotting. The phosphorylation level of ERK1/2 was increased in cells incubated with TGF-β1. The increased phosphorylation level of ERK1/2 was down-regulated by cGMP in 10 ng/mL. This down-regulated effect could be blocked by PKG inhibitor (Figure 5). To study whether there were cross talk effects of PKC inhibitor and cGMP-ERK1/2 pathway, cells were incubated with a culture medium containing the PKC inhibitor and cGMP together. The phosphorylation level of ERK1/2 was also increased significantly by PKC inhibitor (*p* < 0.05, *vs.* cGMP group). It is suggested that in the absence of PKC activation or inhibited PKC activity, the effect of cGMP down-regulated phosphorylation level of ERK1/2 was blocked.

### 2.6. Involvement of Different Subtypes of PKC in Cell Proliferation Regulated by cGMP

**Figure 6:**
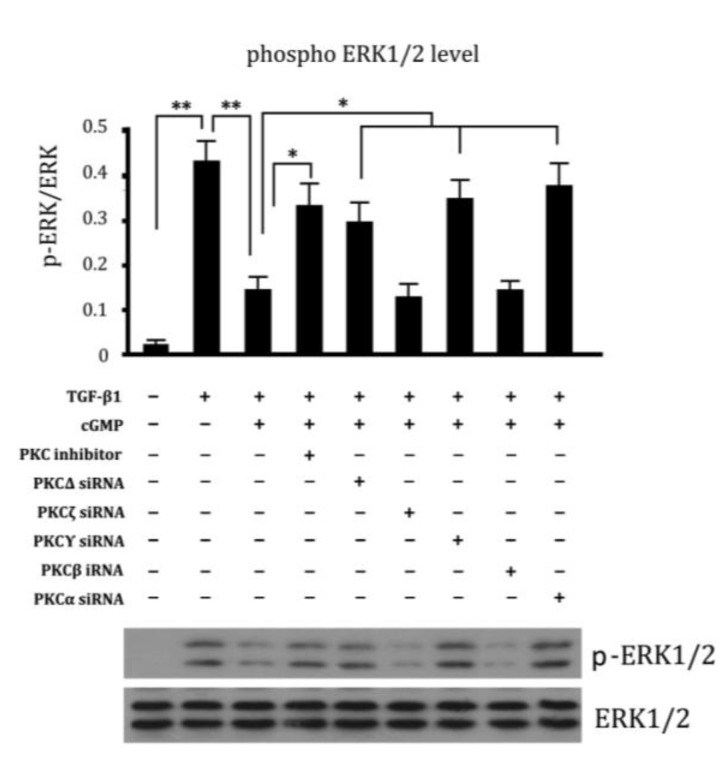
The different subtypes of PKC were studied. The groups were marked as following: 1, Cell; 2, TGF-β1 (10 ng/mL); 3, TGF-β1 (10 ng/mL) + Cgmp (100 ng/mL); 4, TGF-β1 (10 ng/mL) + Cgmp (100 ng/mL) + PKC inhibitor; 5, TGF-β1 (10ng/mL) + Cgmp (100 ng/mL) + PKCΔ siRNA; 6, TGF-β1 (10 ng/mL) + cGMP (100 ng/mL) + PKCζ siRNA; 7, TGF-β1 (10 ng/mL) + cGMP (100 ng/mL) + PKCγ siRNA; 8, TGF-β1 (10 ng/mL) + cGMP (100 ng/mL) + PKCβ siRNA; 9, TGF-β1 (10 ng/mL) + cGMP (100 ng/mL) + PKCα siRNA. ** *p* < 0.01 and * *p* < 0.05.

Since there are various subtypes of PKC existing in cells, we also analyzed which is involved in ERK phosphorylation. It was shown that siRNA targeting PKCΔ, PKCγ and PKCα siRNA eliminated the effect of cGMP down-regulated phosphorylation level of ERK1/2 in cells (Figure 6), suggesting their involvement in this process.

### 2.7. Endothelial Tube Formation Assay

**Figure 7:**
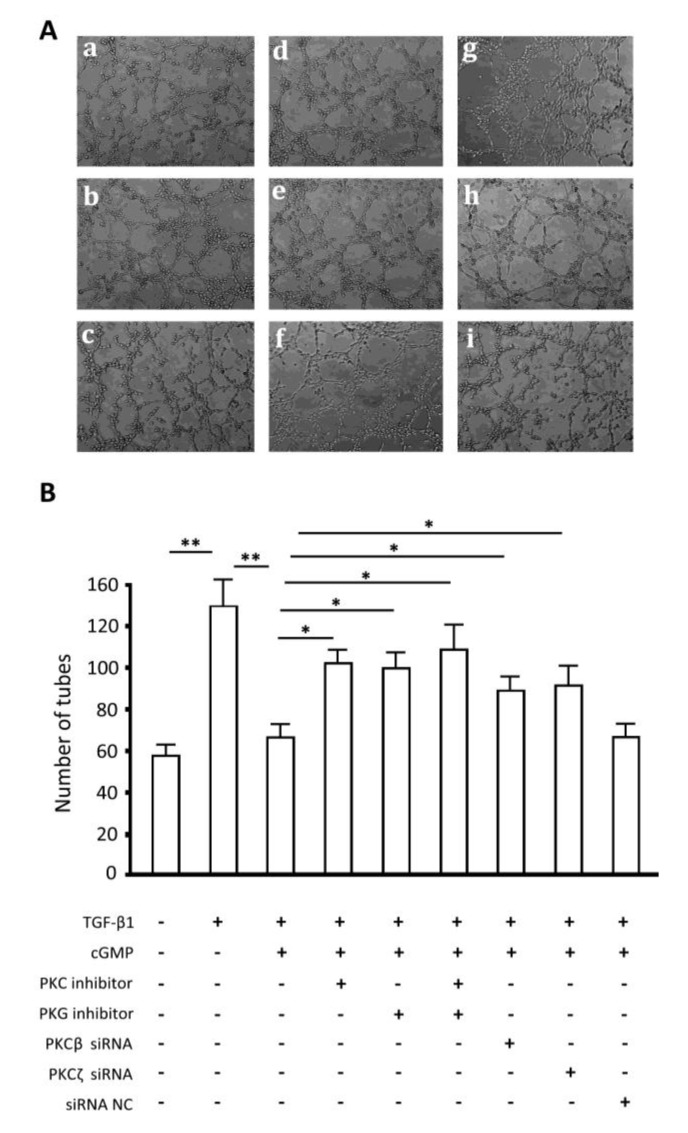
Endothelial tube formation assay. The groups were marked as following: (**A**): (**a**) cell; (**b**) TGF-β1 (10 ng/mL); (**c**) TGF-β1 (10 ng/mL) + cGMP (100 ng/mL); (**d**) TGF-β1 (10 ng/mL) + cGMP (100 ng/mL) + PKC inhibitor; (**e**) TGF-β1 (10 ng/mL) + cGMP (100 ng/mL) + PKG inhibitor; (**f**) TGF-β1 (10 ng/mL) + cGMP (100 ng/mL) + PKC inhibitor + PKG inhibitor; (**g**) TGF-β1 (10 ng/mL) + cGMP (100 ng/mL) + PKCβ siRNA; (**h**) TGF-β1 (10 ng/mL) + cGMP (100 ng/mL) + PKCζ siRNA; and (**i**) TGF-β1 (10 ng/mL) + cGMP (100 ng/mL) + siRNA NC; (**B**) The numbers of complete tubes were analyzed. ** *p* < 0.01 and * *p* < 0.05.

TGF-β1 enhanced tube formation by endothelial cells, as well as extraordinary proliferation; cGMP inhibited the effects on proliferation of TGF-β1, thus impaired tube formation; PKC or PKG inhibitor reduced the effects of cGMP significantly; and there was no obvious difference between results of the combination of PKC and PKG inhibitors and the inhibitors used alone (Figure 7A,B). Moreover, results showed that siRNA-mediated silencing of PKCβ and PKCζ also significantly reduced the effects of cGMP.

### 2.8. Discussion

In this study, we first confirmed cGMP decreased TGF-β1 induced proliferation and tubulation of pulmonary vascular endothelial cells. siRNA-mediated ERK gene silencing clearly revealed an important role that ERK activation plays in TGF-β1 induced migration and proliferation. Then, we investigated the signaling pathways involved in forming tube-like structures of pulmonary vascular endothelial cells. We found the inhibitory effect on tubulation of endothelial cells by cGMP can be blocked by both broad-spectrum inhibitors of PKCs and inhibitors of PKGs, and further, that this effect in the cGMP-PKG pathway is dependent on phosphorylation of ERK.

It seems very difficult to discern an unequivocal role of angiogenesis in the development or progression of PAH. Experimental intervention studies do not resolve this issue: in some studies, blocking angiogenesis was associated with improvement in hypoxic PAH [11], while in other studies, stimulation of angiogenesis ameliorated hypoxic PAH [12]. Also, there is an ongoing controversy as to whether chronic hypoxia leads to loss of pulmonary vasculature [13]. In tube formation experiments of endothelial cells derived from EPCs, we found that cGMP inhibited tube formation of endothelial cells, which may be adverse to pulmonary vascular repair. However, based on the controversial roles of EPCs in pulmonary arterial hypertension, the results may be interpreted further. In general, endothelial cells undergo apoptosis after blood vessel injuries. In the process of apoptosis, some endothelial cells may develop resistance to apoptosis, and proliferate continuously, which causes the malformation of vessel and increases pressure. Due to the response of cells to this change, intracellular cGMP accumulates and inhibits excess proliferation of endothelial cells. The problem is that some endothelial cells of normal functions may be damaged inevitably in this process, which is adverse to vascular repair. Interestingly, cGMP does not induce significant apoptosis in endothelial cells while inhibiting their proliferation. In tube formation assay in intro, we found that the key was that during inhibition of excessive proliferation of cells resistant to apoptosis, cGMP also enabled endothelial cells derived from endothelial progenitor cells to form tube rapidly, so it did not induce significant apoptosis in regenerative endothelial cells. Clearly, further study is also required to elucidate the detailed effects of cGMP on genesis and development of PAH.

In accordance with the research in treatment of pulmonary hypertension with sildenafil [14], the traditional cGMP-NO signal pathway not only reduces pulmonary vasoconstriction, but also affects the pulmonary vascular remodeling. The possible mechanisms include down-regulation of RhoA expression [15], leading to the abolition of RhoA/Rho kinase-mediated Ca^2+^ sensitization of contraction, which is responsible for the decreased responses to contracting agonists in the pulmonary artery of chronic hypoxia (CH) rats. A more direct effect is caused by down-regulation of ERK phosphorylation mediated by the cGMP-PKG pathway [6,7], which suppresses proliferation of pulmonary vascular endothelial cells, dependent on ERK phosphorylation. But in this study, we confirmed cGMP incubation significantly reduced tubulation in pulmonary vascular endothelial cells, which was related to the cGMP-PKG pathway. Similar to PKG inhibitors, PKC inhibitor blocked the inhibitory effect of cGMP on cell tabulation, which is dependent on ERK activation, as mentioned earlier.

As an important cellular signal transduction molecule, PKCs mediate a variety of cellular responses, such as cell secretion, gene expression, cell proliferation, and muscle contraction [16,17]. Previously, Tan *et al.* [9] showed that in a bird model of pulmonary hypertension caused by cold stimulation, PKCα expression was increased with pulmonary hypertension, involved in pulmonary vascular remodeling. It is shown that PKC regulates ERK activation in a complex way. When conducting different signals from the cell surface, PKC protein regulates ERK activation via Ras-independent as well as Ras-dependent pathways [18,19]. Additionally, there may be MAPKK (mitogen-activated protein kinase kinase)-independent routes of PKC-dependent ERK activation, including direct phosphorylation of ERK by PKC [20].

**Figure 8:**
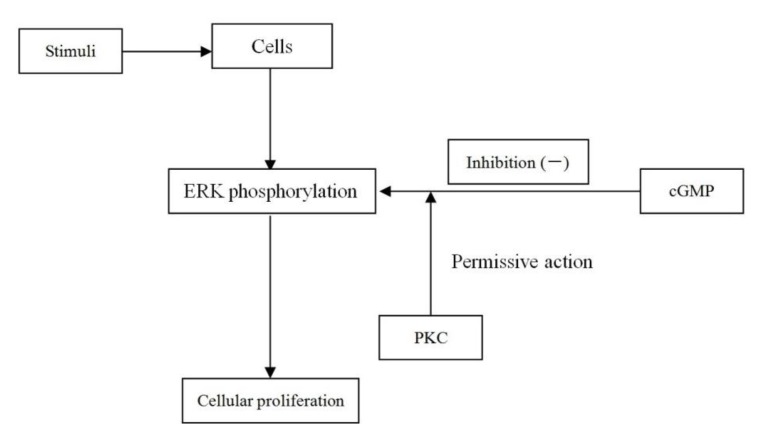
The algorithm showing the permissive action of PKC.

The present study was to investigate what roles ERK phosphorylation plays in pulmonary vascular endothelial cell and how it eventually affects pulmonary vascular remodeling. Interestingly, and different to the aforesaid studies, results from ERK phosphorylation measurement showed that cGMP inhibited pulmonary vascular endothelial cell proliferation through impairing ERK phosphorylation, which was blocked by PKG inhibitors. In addition, the broad-spectrum PKC inhibitor exhibited similar effects. A possible mechanism could be, when PKC kinase exists, the inhibition effect of cGMP on ERK phosphorylation is valid, and ERK phosphorylation is a critical factor in pulmonary vascular endothelial cell proliferation. In other words, PKC kinase plays permissive action in the inhibition effect of cGMP on ERK phosphorylation in signal transduction of PVEC proliferation. Therefore, the broad spectrum PKC inhibitor exhibits not only an effect to facilitate the pulmonary vascular endothelial cell proliferation, but an effect to abrogate suppressed ERK phosphorylation by cGMP (as shown in Figure 8).

Further, silencing different PKC isoforms, we found that PKCΔ, PKCγ and PKCα are involved in ERK phosphorylation. As PKCγ and PKCα activate in a calcium dependent way and PKCΔ is a calcium-independent subtype, PKCs may suppress ERK phosphorylation through the cGMP-PKG pathway provided different stimuli as a kinase having a permissive action, (whether calcium-dependent or calcium-independent conditions), ultimately to attenuate cell proliferation and pulmonary vascular remodeling.

However, it has not been elucidated whether a substrate is involved in the process whereby PKC inhibits ERK phosphorylation via the cGMP-PKG pathway. PKC has been considered to be active under basal conditions, and to phosphorylate Thr495 of eNOS (endothelial nitric oxide synthase) to inactivate the enzyme and consequently inhibit NO synthesis [21]. Inhibition of NO synthesis is considered to be one of the reasons for pulmonary vascular remodeling; why PKC kinases play different roles in pulmonary vascular remodeling need further study.

## 3. Experimental Section

### 3.1. Antibody and Regents

Rat recombinant TGF-β1 was purchased from PeproTech (Rocky Hill, MI, USA). Lipofectamine 2000, 0.25% trypsin, fetal bovine serum (FBS) and RPMI-1640 were purchased from Invitrogen (Carlsbad, MD, USA). The rabbit anti rat polyclonal antibodies against ERK, phospho-ERK and SMA (α-smooth muscle actin) were purchased from Cell Signaling Technology (Boston, MA, USA). Consumables for cell culture were obtained from Corning (Rosemont, NY, USA). MTT and DMSO (Dimethy1 sulfoxide) were purchased from Sigma (Saint Louis, WY, USA). PKC inhibitor and PKG inhibitor were purchased from Santa Cruz. All other reagents were obtained from local commercial sources.

### 3.2. Cell Culture

#### 3.2.1. Isolation and Culture of Rat Pulmonary Microvascular Endothelial Cells

After obtaining approval from the Animal Care and Use Committee at the 6th Hospital affiliated to Shanghai Jiaotong University, pulmonary vascular endothelial cell were isolated by means of the explant culture method. Isolation and culture of PVEC has been described previously [22]. Cells were maintained under static culture conditions before being subjected to flow. Rat pulmonary microvascular endothelial cells (RPMVESs) at passage 3 were used in the experiments.

#### 3.2.2. Isolation and Identification of Rat Peripheral Blood Endothelial Progenitor Cells and Differentiation of Endothelial Cells

Mononuclear cells were isolated from 10 mL arterial blood extracted from a SD (Sprague Dawley) rat and mixed with 10 mL IMDM (Iscove’s Modified Dulbecco’s Media) by Ficoll density gradient centrifugation, and seeded to fibronectin coated culture plates, and incubated at 37 °C in a 50 mL/L CO_2_ air incubator. IMDE (containing 100 mL/L fetal calf serum, 100 mL/L horse serum, 30 μL/mL VEGF (vascular endothelial growth factor) and 10 μL/mL bFGF (basic fibroblast growth factor)) was used and changed every 3 days. The adherent cells reached almost confluency on day 10. Immumohistochemical staining showed positive expression of endothelial cell’s markers, CD31, CD34, Flk21, and vMF, one week after seeding, suggesting that EPCs differentiated into endothelial-like cells under the inducing environment. The cells were used for following *in-vitro* experiments.

### 3.3. Cell Treatment

#### 3.3.1. Treatment A

The culture medium was changed to serum-free RPMI-1640 medium 12 h after seeding and the cells were cultured under normal conditions for another 6 h, and TGF-β1 was added to the culture medium to final concentrations of 1, 10 and 100 ng/mL, and the cells were cultured for another 24 or 48 h, and MTT assay was conducted to analyze the effects of TGF-β1 on PVECs proliferation (concentration and time-dependence), to determine appropriate conditions of the protein factor for following experiments.

#### 3.3.2. Treatment B

The culture medium was replaced with serum-free RPMI-1640 medium 12 h after seeding and the cells were cultured under normal conditions for another 6 h, cGMP was added into the medium to a final centration of 10 ng/mL; after 4 h of incubation, TGF-β1 was added to the culture medium to a final concentration of 100 ng/mL, and the cells were cultured for another 48 h, and MTT assay was conducted to analyze the effects of cGMP on PVECs proliferation and on cellular proliferation induced by TGF-β1.

#### 3.3.3. Treatment C

The culture medium was changed to serum-free RPMI-1640 medium 12 h after seeding and the cells were cultured under normal conditions for another 6 h, PKG inhibitor or PKC inhibitor was added into the medium to a final centration of 100 ng/mL; after 4 h of incubation, cGMP was added into the medium to a final centration of 10 ng/mL; TGF-β1 was added to the culture medium to a final concentration of 100 ng/mL, and the cells were cultured for another 48 h, and MTT assay was conducted to analyze the effects of the two inhibitors on changes in cellular proliferation induced by cGMP.

#### 3.3.4. Treatment D

The culture medium was replaced with serum-free RPMI-1640 medium 12 h after seeding and the cells were cultured under normal conditions for another 6 h, cGMP was added into the medium to a final centration of 10 ng/mL; for the group of genetic intervention, siRNA-ERK1 and siRNA2 was transfected, and the cells were cultured for another 48 h, and MTT assay was conducted to analyze the effects of ERK on changes in cellular proliferation induced by cGMP.

#### 3.3.5. Treatment E

Cells were seeded into 6-well cell culture plate, 2 mL per well. Cells were treated with cGMP (100 ng/mL), PKC inhibitor (50 ng/mL), PKG inhibitor (50 ng/mL) alone or in combination for 2 h. Then, TGF-β1 was added into the medium to a final concentration of 10 ng/mL, and the cells were collected after an incubation of 24 h. Total protein was extracted and ERK 1/2 phosphorylation was detected by western blotting.

### 3.4. MTT (3-4,5-Dimethyl-2-thiazolyl)-2,5-diphenyl-2-H-tetrazolium bromide) Assay

MTT assay was employed to detect cell viability. PBS (phosphate buffered saline) (0.1 mL) and 10 μL MTT were added to each well and the cells were incubated for another 4 h at 37 °C; the supernatant was removed, and 0.1 mL dimethyl sulfoxide was added to each well. The plates were shaken for 15 min (80 r/min, 37 °C) and the optical density (OD) values were determined at a wavelength of 490 nm by an enzyme-linked detector (Thermo, Waltham, MA, USA).

### 3.5. Design of siRNA Targeting ERK1/2 Genes

Three different sequences targeting Rat ERK mRNA were chosen based on the full-length mRNA sequence of Rat ERK1/2 in Genbank (Accession No.: NM_017347.2/NM_053842.1). The target sequences of ERK1 and ERK2 were 5'-GCCAUGAGAGAUGUUUACA-3' and 5'-GCUCUUGAAGACACAGCAC-3'. The synthesized siRNAs and their complementary sequence (Invitrogen, Shanghai, China) were diluted in DEPC, and were annealed before transfection. Transfection was carried out following the manufacturer’s instructions for Lipofectamine 2000.

### 3.6. Western Blotting

Total protein samples were extracted from the treated cells and quantified using the bicinchoninic acid protein assay kit (Pierce, Rockford, IL, USA). Samples were denatured at 95 °C for 10 min, separated by SDS-PAGE at a separation gel concentration of 12% and transferred to PVDF membranes (Millipore, Billerica, MA, USA) at a constant current (400 mA). The blots were blocked in TBST containing 5% nonfat milk at room temperature for 2 h and incubated with the primary antibodies (dilution: ERK1/2, 1:800; VP-ERK1/2, 1:500) at 4 °C overnight, and then incubated with secondary antibodies (1:3000). The bands were developed by ECL chemiluminescence substrates (Thermo) using X-ray films and were scanned and quantified by densitometric analysis.

### 3.7. In Vitro Tube Formation Assay

Pretreatment of cells: PVECs at logarithmic phase were made into single cell suspension by trypsinization and seeded to 6-well plates, and pretreated with PKC inhibitor, PKCζ siRNA and PKCβ iRNA for 24 h. The cells were incubated for 4 h after the medium was replaced with the medium containing 100 ng/mL cGMP. Then, TGF-β1 was added to a final concentration of 10 ng/mL, and the cells were incubated for 20 h under normal conditions.

Tube formation assay: 96-well plates and tips were incubated at −20 °C for 30 min and Matrigel (BD) was transferred onto ice. Matrigel (10 μL) was added to each well with the cool tip and distributed evenly by shaking the plate. The plate was placed on ice for 2 min and then incubated in a cell culture incubator for 30 min. Cell suspension was made by trypsinization and adjusted to 1 × 10^5^ cells/mL after live cell counting. Cells were added into the pre-incubated plate at 100 μL/well, and incubated under normal conditions. Images were acquired on an inverted fluorescence microscopic 6 h later. Then the numbers of complete tubes from five randomly chosen fields were counted and analyzed. Each group consisted of three or five Matrigels.

### 3.8. Statistics

All experiments were repeated at least three times in quintuplicate or more (*n* ≥ 5). The data were expressed as mean ± SEM. ANOVA (analysis of variance) was used to determine the statistical significance of the data, followed by Tukey post hoc test. Difference with *p* < 0.05 was considered statistically significant.

## 4. Conclusions

In conclusion, cGMP inhibits pulmonary endothelial cells to form tube-like structures induced by TGF-β1, which is mediated through ERK phosphorylation; PKC kinases have a permissive action, and three subtypes, PKCΔ, PKCγ and PKCα are likely to be involved.
